# Supplementary material for: Beyond malaria prevention: sulfadoxine-pyrimethamine treatment in pregnancy selectively remodels the maternal gut microbiome to increase gestational weight gain and improve birthweight
Source: medRxiv. 2026 May 5:2026.05.03.26352319. Preprint. [Version 1] doi: 10.64898/2026.05.03.26352319 (PMC13174731; doi:10.64898/2026.05.03.26352319)
Supplement: Supplement 3 [file media-3.pdf]

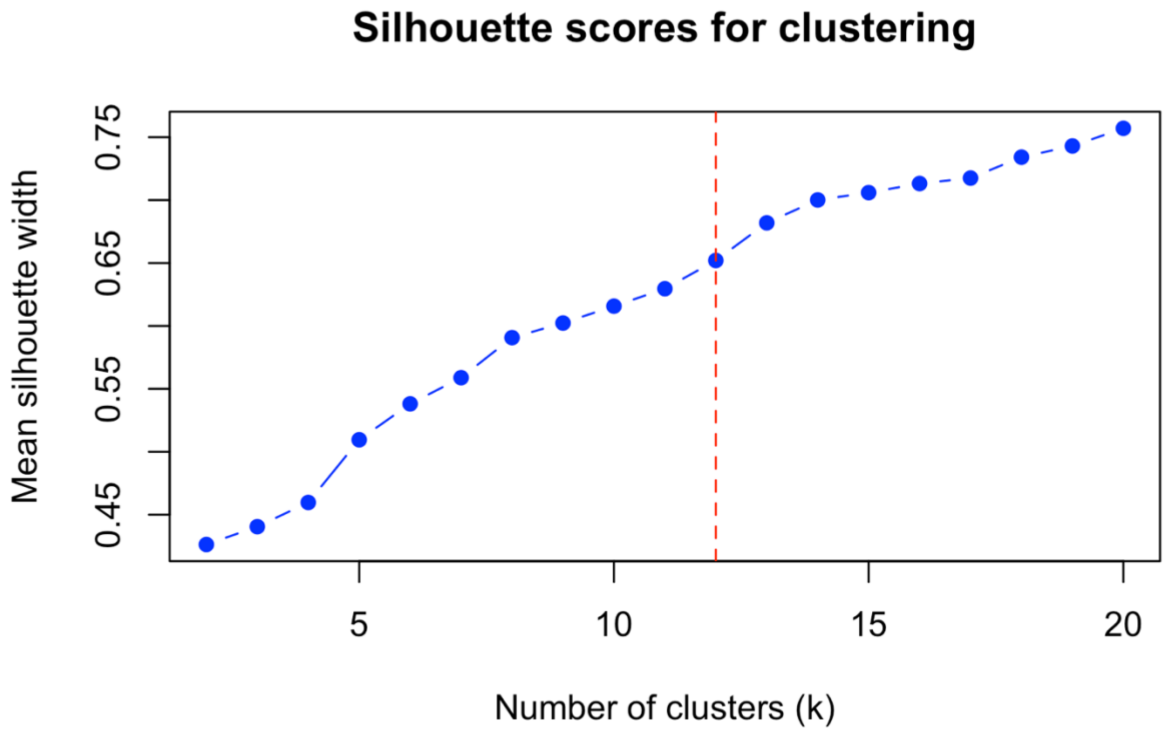

Extended Data Fig. 3. Silhouette score evaluation across k=2-20 for hierarchical clustering of 1,152 isolate genomes by folate pathway gene presence/absence (Ward's method, Manhattan distance). Mean silhouette width increases across the range evaluated, consistent with the binary, low-dimensional nature of the 15-gene presence/absence matrix in which finer partitioning continues to improve within-cluster homogeneity without producing a clear inflection point. In this setting, silhouette maximization does not identify a single optimal k; instead, k=12 (red dashed line) was selected on the basis of parsimony and biological interpretability.
