# Supplementary material for: Beyond malaria prevention: sulfadoxine-pyrimethamine treatment in pregnancy selectively remodels the maternal gut microbiome to increase gestational weight gain and improve birthweight
Source: medRxiv. 2026 May 5:2026.05.03.26352319. Preprint. [Version 1] doi: 10.64898/2026.05.03.26352319 (PMC13174731; doi:10.64898/2026.05.03.26352319)
Supplement: Supplement 6 [file media-6.pdf]

Table 1. Baseline characteristics of the ancillary microbiome cohort by randomized group

Characteristic	Overall	IPTp-SP	IPTp-DP
Participants, n	91	45	46
**Maternal demographics**			
Age at enrollment, years	20.9 (2.6)	20.8 (2.5)	20.9 (2.7)
**Gravidity category**			
1	41 (45.1%)	22 (48.9%)	19 (41.3%)
2	34 (37.4%)	15 (33.3%)	19 (41.3%)
3	16 (17.6%)	8 (17.8%)	8 (17.4%)
**Wealth tertile**			
1	43 (47.3%)	19 (42.2%)	24 (52.2%)
2	25 (27.5%)	13 (28.9%)	12 (26.1%)
3	23 (25.3%)	13 (28.9%)	10 (21.7%)
**Anthropometry**			
Weight at enrollment, kg	56.3 (7.2)	56.7 (7.6)	55.9 (6.8)
MUAC at enrollment, cm	25.8 (2.2)	25.7 (2.1)	26.0 (2.4)
Height, cm	157.3 (5.6)	157.8 (6.1)	156.9 (5.0)
BMI at enrollment, kg/m^2	22.9 (2.7)	22.9 (2.4)	23.0 (2.9)
**Clinical**			
Malaria PCR positive at enrollment	27 (29.7%)	14 (31.1%)	13 (28.3%)

Values are mean (SD) or n (%). Percentages are calculated among participants with available data. No statistical tests were performed for baseline comparisons between randomized groups.
